# Deletion of *Akt1* Promotes Kidney Fibrosis in a Murine Model of Unilateral Ureteral Obstruction

**DOI:** 10.1155/2020/6143542

**Published:** 2020-11-24

**Authors:** Il Young Kim, Min Young Lee, Mi Wha Park, Eun Young Seong, Dong Won Lee, Soo Bong Lee, Sun Sik Bae, Sang Soo Kim, Sang Heon Song

**Affiliations:** ^1^Research Institute for Convergence of Biomedical Science and Technology and Department of Internal Medicine, Pusan National University Yangsan Hospital, Yangsan, Republic of Korea; ^2^Biomedical Research Institute, Pusan National University Hospital, Busan, Republic of Korea; ^3^Department of Internal Medicine, Pusan National University Hospital, Busan, Republic of Korea; ^4^MRC for Ischemic Tissue Regeneration, Medical Research Institute, and Department of Pharmacology, Pusan National University School of Medicine, Yangsan, Republic of Korea

## Abstract

We investigated the role of Akt1, one of the three isoforms of Akt, in renal fibrosis using the murine model of unilateral ureteral obstruction (UUO). We subjected wild type and *Akt1*^−/−^ mice to UUO. The Akt1 gene was silenced in vitro using short hairpin RNA delivered via a lentiviral vector in human proximal tubular cells (HK2 cells) and kidney fibroblasts (NRK-49F cells). The obstructive kidneys of *Akt1*^−/−^ mice showed more severe tubulointerstitial fibrosis than those of wild type mice. The expression of fibronectin and type I collagen was significantly increased in obstructed kidneys of *Akt1*^−/−^ mice compared to those of wild type mice. The important finding was that the expression of transforming growth factor *β*1 (TGF*β*1) was significantly increased in the *Akt1*^−/−^ mice compared to the wild type mice. The knockdown of *Akt1* enhanced the expression of TGF*β*1 in HK2 cells. Interestingly, the upregulation of TGF*β*1 due to genetic knockdown of *Akt1* was associated with activation of signal transducer and activator of transcript 3 (STAT3) independently of the Smad pathway in NRK-49F and HK2 cells. Immunohistochemical staining also showed that expression of phosphorylated STAT3 was more increased in *Akt1*^−/−^ mice than in wild type mice after UUO. Additionally, the deletion of *Akt1* led to apoptosis of the renal tubular cells in both in vivo and in vitro studies. Conclusively, these results suggest that the deletion of *Akt1* may contribute to renal fibrosis via induction of the TGF*β*1/STAT3 pathway in a murine model of UUO.

## 1. Introduction

Chronic kidney disease (CKD) is a highly prevalent disease, which is caused by multiple etiologies including diabetes mellitus (DM), hypertension, and glomerulonephritis. Accordingly, CKD imposes a heavy socioeconomic burden. The prevalence of CKD varies between 7% and 12% in different regions of the world [1], and the Korean National Health and Nutritional Examination Survey (2011-2013) reported a prevalence of 8.2% in adults aged ≥20 years [2].

Renal fibrosis is a common characteristic of CKD and is referred to as “glomerulosclerosis” or “tubulointerstitial fibrosis” according to its anatomic location [3, 4]. Furthermore, these findings are usually accompanied by peritubular capillary rarefaction, interstitial inflammation, and tubular atrophy [3]. Also, tubular apoptosis can importantly contribute to renal fibrosis [5].

Akt/protein kinase B (PKB) is a serine/threonine kinase with three isoforms (Akt1, 2, and 3), which are all activated by phosphatidylinositide 3′-OH kinase (PI3K) and participate in various cellular signaling processes including glucose metabolism, apoptosis, and cell proliferation and migration [6, 7]. In cancer cells, Akt has an antiapoptotic action and has been proposed to act along with PI3K as a mediator of cell survival [8]. Previous studies using various transgenic and knockout (KO) mice have shown that Akt isoforms have partly redundant but also distinct functions in physiological and pathological processes, in part due to different tissue-specific expressions of the isoforms [9–11]. The activity of Akt is increased in the kidneys in experimental tubulointerstitial fibrosis [7]. However, the role of the Akt isoforms in tubulointerstitial fibrosis remains to be determined. Akt isoforms may have different functions and can induce different phenotypes in a cell- and organ-dependent manner [10, 11]. Moreover, we previously reported that the genetic silencing of Akt1 causes the vascular smooth muscle cells to switch from the contractile to the synthetic phenotype indicating that Akt1 could be related to the fibrosis pathway [12]. The unilateral ureteral obstruction (UUO) model is a well-established model that exhibits all the features of CKD. Thus, in this study, we investigated the role of Akt1, one of the three Akt isoforms, in renal fibrosis and tubular apoptosis using the murine model of UUO.

## 2. Materials and Methods

### 2.1. Murine Models of Renal Fibrosis

The protocols (PNU 2015-0909, 0998) for animal use were reviewed and approved by Pusan National University–Institutional Animal Care and Use Committee (PNU-IACUC) with respect to ethics and husbandry. Male wild type mice (C57BL/6) were purchased from Koatech Technology Corporation (Seoul, Korea), and male mice lacking *Akt1* (*Akt1*^−/−^) (C57BL/6, 129P2-*Akt1^tm1Mbb^*/J) were purchased from Jackson Laboratory (Bar Harbor, Maine, USA). All mice were housed in cages under a 12-hour light/dark cycle, and the temperature was maintained at 22°C. Additionally, the mice were fed the standard laboratory diet and allowed free access to water. All procedures were conducted in the same way for both the groups and conformed to the Guidelines for the Care and Use of Experimental Animals endorsed by the Korean Society of Experimental Animal. The mice were euthanized via 2% isoflurane inhalation followed by exsanguination and cardiac perfusion using phosphate-buffered saline. For UUO, 9-week-old wild type and *Akt1*^−/−^ mice were anesthetized with 2% isoflurane. In each animal, the left kidney was exposed through a left flank incision, and the ureter was then exposed by gentle dissection and ligated with a 6-0 silk tie suture at two points. The mice were euthanized 1, 3, or 7 days after surgery. Whole kidneys were harvested, and one-half of each kidney was fixed in 10% neutralized formalin for immunohistochemistry. The samples were identified as follows: A1 (*n* = 6): day 1 *Akt1*^−/−^, A3 (*n* = 6): day 3 *Akt1*^−/−^, and A7 (*n* = 6): day 7 *Akt1*^−/−^, and U1 (*n* = 6): day 1 wild type, U3 (*n* = 6): day 3 wild type, and U7 (*n* = 6): day 7 wild type. The wild type control sham (U0, *n* = 6) and *Akt1*^−/−^ control sham (A0, *n* = 6) mice were subjected to identical surgical procedures without occlusion of the ureter.

### 2.2. Microscopic Analysis/IHC

Immediately after collection, the tissues were fixed in 10% formalin and processed and embedded in paraffin. For Masson's trichrome staining, 4 *μ*m thick sections of paraffin-embedded tissues were deparaffinized, rehydrated in an ethanol series, washed with tap water, and refixed in Bouin's solution (HT10132, Sigma-Aldrich, Saint Louis, MO, USA) overnight at room temperature. After washing with running tap water for 10 min, the sections were stained with Weigert's iron hematoxylin working solution for 10 min. The sections were then stained with Biebrich scarlet-acid fuchsin solution for 10 min, washed for 10 min, treated with a phosphomolybdic-phosphotungstic acid solution (Sigma-Aldrich, Saint Louis, MO) for 10 min, transferred to aniline blue solution for 7 min, and then reacted with 1% acetic acid solution for 1 min.

IHC was performed on formalin-fixed 3 *μ*m thick paraffin-embedded sections. Briefly, the sections were deparaffinized, rehydrated using an ethanol series, blocked with normal horse serum, treated with primary antibodies at 4°C overnight, and incubated for 30 min with a secondary antibody (ImmPRESS™ HRP reagent kit, Vector Laboratories, CA, USA). Finally, the slides were developed using DAB (Vector Laboratories, CA, USA) and counterstained with Harris hematoxylin. The following antibodies were used for IHC: anti-fibronectin antibody diluted 1 : 250 (ab2413, Abcam, Cambridge, UK), anti-type I collagen antibody diluted 1: 200 (ab34710, Abcam, Cambridge, UK), anti-tumor growth factor *β*1 (TGF*β*1) antibody diluted 1 : 100 (ab92486, Abcam, Cambridge, UK), anti-cleaved caspase-3 antibody diluted 1 : 150 (#9661, Cell Signaling Technology, Boston, MA, USA), and anti-phosphorylated- (p-) signal transducer and activator of transcript 3 (STAT3) antibody diluted 1 : 100 (9139/9131, Cell Signaling Technology, Boston, MA, USA). Masson's trichrome staining was performed to analyze the degree of kidney fibrosis. Scans of the stained tissue were obtained using digital Aperio Scanscope CS2 (Leica Biosystems, Nußloch, Germany). Digital images of at least 10 cortical fields (×200) were examined by Aperio ImageScope v.12. Glomeruli and large vessels were not included in the image analysis, and subcapsular and perivascular areas were excluded from quantification. Type I collagen and fibronectin were quantified in the same manner. Additionally, cleaved caspase-3 staining was quantified as the numbers of positive nuclei per 100 cross-sectioned tubules in a high-power field (×200). The p-STAT3 staining was quantified as the numbers of positive cells per 100 tubules in 5 randomly chosen (0.4 × 0.4 mm^2^) tubulointerstitial areas.

### 2.3. In Vitro Cell Culture of Two Different Cell Lines

Immortalized human proximal tubular cells (HK2 cells) were obtained from ATCC (Manassas, VA, USA) and cultured in Dulbecco's modified Eagle's medium (DMEM) containing 4.5 g/L D-glucose, 10% heat-inactivated fetal bovine serum, and penicillin-streptomycin (Life Technologies, Paisley, UK). Rat kidney fibroblasts (NRK-49F cells) were obtained from ATCC (Manassas, VA, USA) and cultured in DMEM containing 4.5 g/L D-glucose, 5% fetal bovine serum, and penicillin-streptomycin. Both the cell lines were incubated at 37°C in a humidified atmosphere at 5% CO_2_/95% air. Cells were starved with 1% FBS for 24 hours before experiments. After that, we treated TGF*β*1 (10 ng/mL) and angiotensin II (1 *μ*M) for 24 hours after switching to fresh media with 1% FBS.

### 2.4. Short Hairpin RNA and Constructs

To silence Akt1, a *shAkt1* (5′-CGA GTT TGA GTA CCT GAA GCT-3′) oligonucleotide with an *AgeI* site at the 5′ end and an *EcoRI* site at the 3′ end was designed, and the sense and antisense oligonucleotides were synthesized (XenoTech, Daejeon, Korea). Both complementary oligonucleotides were mixed, heated at 98°C for 5 min, and cooled to room temperature. The annealed nucleotides were subcloned into the *AgeI/EcoRI* sites of the pLKO.1 lentiviral vector.

### 2.5. Lentiviral Knockdown

Lentiviral knockdown was performed as described previously [12]. For gene silencing, HEK293-FT packaging cells (Invitrogen) were grown to ~70% confluency in 6-well plates and triple transfected with 6 *μ*g of PRO-PREP™ (iNtRON Biotechnology), 1 *μ*g of *Δ*8.9, and 1 *μ*g of pVSV-G by calcium phosphate method. The medium was replaced with fresh medium at 8 h after transfection. Lentiviral supernatants were harvested at 24 h posttransfection and filtered using 0.45 *μ*m filters. Cell-free viral culture supernatants were used to infect HK2 and NRK-49F cells in the presence of 8 *μ*g/mL of polybrene (Sigma-Aldrich, Saint Louis, MO, USA). An additional round of transfection was performed at 48 and 72 h after initial transfection. Infected cells were isolated by puromycin selection after 2 days of exposure to 10 *μ*g/mL puromycin.

### 2.6. Western Blotting

Western blotting was performed as described previously [13]. Proteins were extracted from cells using a protein extraction solution (PRO-PREP™, iNtRON Biotechnology, Korea), and protein concentrations were measured by the Bradford method (Bio-Rad Protein Assay; Bio-Rad Laboratories Inc., Hercules, CA, USA). Proteins were separated by electrophoresis on 12% SDS-polyacrylamide gels and transferred onto nitrocellulose membranes (Hybond ECL, Amersham Pharmacia Biotech Inc., Piscataway, NJ, USA), which were blocked for 2 h at room temperature with 5% (*w*/*v*) nonfat dried milk in Tris-buffered saline (10 mM Tris/HCl, pH 8.0, and 150 mM NaCl) containing 0.05% Tween-20. The membranes were immunoblotted with the following specific primary antibodies: anti-TGF*β*1 antibody diluted 1 : 500 (ab190503, Abcam, Cambridge, UK), anti-Akt1/Akt2 antibody diluted 1 : 2,000 (07-416/07-372, Millipore, MA, USA), anti-fibronectin antibody diluted 1 : 1,000 (ab2413, Abcam, Cambridge, UK), anti-type I collagen antibody diluted 1 : 1,000 (ab34710, Abcam, Cambridge, UK), anti-cleaved caspase-3 antibody diluted 1 : 1,000 (9661, Cell Signaling Technology, Boston, MA, USA), anti-Bax antibody diluted 1 : 100 (Santa Cruz Biotechnology, USA), anti-Bcl2 diluted 1 : 100 (Santa Cruz Biotechnology, USA), GAPDH diluted 1 : 2,000 (Santa Cruz Biotechnology, USA), *β*-actin diluted 1 : 1,000 (Santa Cruz Biotechnology, USA), anti-Smad2/Smad3/Smad7 antibody diluted 1 : 500 (511300/511500/420400, Thermo Fisher Scientific, Waltham, MA, USA), anti-p-Smad2 antibody diluted 1 : 1,000 (Cell Signaling Technology, Boston, MA, USA), anti-p-Smad3 antibody diluted 1 : 1,000 (600-401-919, Rockland Immunochemicals, Gilbertsville, PA, USA), and anti-signal transducer and activator of transcript 3 (STAT3)/p-STAT3 antibody diluted 1 : 1,000/1 : 2,000 (9139/9131, Cell Signaling Technology, Boston, MA, USA). The blots were then incubated with the corresponding goat anti-rabbit or goat anti-mouse immunoglobulin G conjugated with horseradish peroxidase diluted at 1 : 4,000 (Enzo Life Science, NY, USA). Immunoreactive proteins were detected using an ECL western blotting detection system (Ab Frontier, Seoul, Korea).

### 2.7. Statistical Analysis

All statistical analyses were performed using GraphPad Prism v.6.0. The Mann–Whitney *U* test or the Kruskal-Wallis test with Dunn's multiple comparison test was used to compare experimental groups according to the duration of UUO, as appropriate. Statistical significance was accepted for *P* values < 0.05, and results are presented as mean ± standard deviation (S.D.).

## 3. Results

### 3.1. The Effect of Akt1 Deletion on Kidney Fibrosis in Obstructed Kidneys of UUO Mice

**Figure 1:**
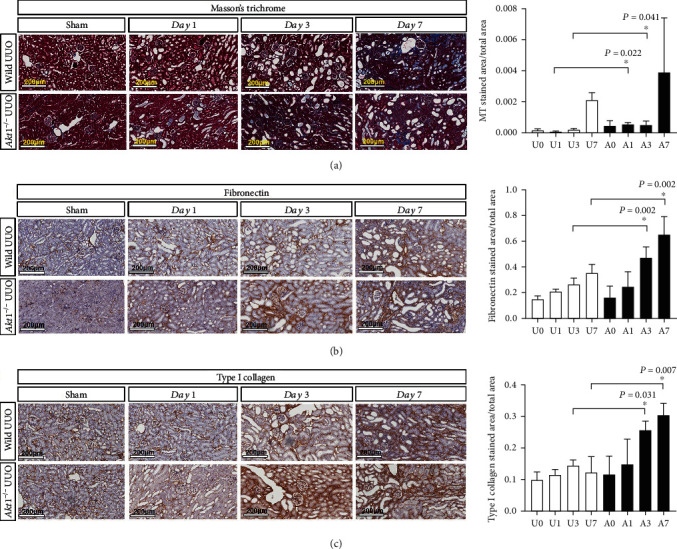
Microscopic comparison of obstructed kidneys between *Akt1*^−/−^ and wild type mice in a murine model of UUO (×200). (a) Masson's trichrome staining. The obstructed kidneys of *Akt1*^−/−^ mice have more sclerotic area than the kidneys of the wild type mice on day 1 (U1 vs. A1, *P* = 0.022) and day 3 (U3 vs. A3, *P* = 0.041) after UUO. (b, c) Immunohistochemical staining. Fibronectin and type I collagen were more strongly expressed in *Akt1*^−/−^ mice than in wild type mice on day 3 (U3 vs. A3; fibronectin, *P* = 0.022; type I collagen, *P* = 0.031) and day 7 (U7 vs. A7; fibronectin, *P* = 0.041; type I collagen, *P* = 0.002) after UUO. Abbreviations: UUO: unilateral ureteral obstruction; A0 (*n* = 6): *Akt1*^−/−^ sham; A1 (*n* = 6): day 1 *Akt1*^−/−^; A3 (*n* = 6): day 3 *Akt1*^−/−^; A7 (*n* = 6): day 7 *Akt1*^−/−^; U0 (*n* = 6): wild type sham; U1 (*n* = 6): day 1 wild type; U3 (*n* = 6): day 3 wild type; U7 (*n* = 6): day 7 wild type. ^∗^*P* < 0.05.

**Figure 2:**
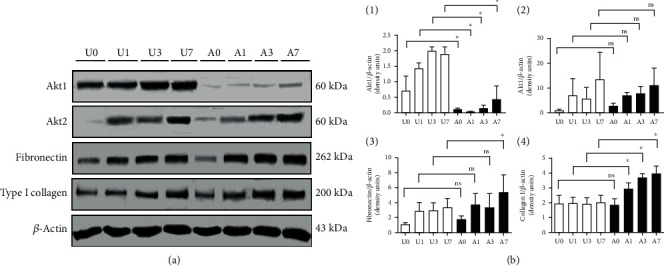
Akt1 is related to proteins associated with tubulointerstitial fibrosis in a murine model of UUO. (a) Western blot analysis. Each kidney of *Akt1*^−/−^ and wild type mice was subjected to western blotting using the indicated antibodies. (b) Quantitative analyses of western blot of Akt1, Akt2, fibronectin, and type 1 collagen were conducted by measurement of optical density using a densitometer. Akt1 was rarely expressed in *Akt1*^−/−^ mice but highly expressed in wild type mice after UUO compared to wild type sham mice. Akt2 expression increased to a similar degree in both the groups after UUO independent of *Akt1* deletion. On day 7 after UUO, *Akt1*^−/−^ mice showed higher levels of fibronectin than wild type mice. Type I collagen was also more strongly expressed in the *Akt1*^−/−^ mice than in the wild type mice on days 1, 3, and 7 after UUO (*P* < 0.05). The images for each indicated probe were cropped from the same blot of the same gel. Abbreviations: UUO: unilateral ureteral obstruction; A0 (*n* = 6): *Akt1*^−/−^ sham; A1 (*n* = 6): day 1 *Akt1*^−/−^; A3 (*n* = 6): day 3 *Akt1*^−/−^; A7 (*n* = 6): day 7 *Akt1*^−/−^; U0 (*n* = 6): wild type sham; U1 (*n* = 6): day 1 wild type; U3 (*n* = 6): day 3 wild type; U7 (*n* = 6): day 7 wild type.

**Figure 3:**
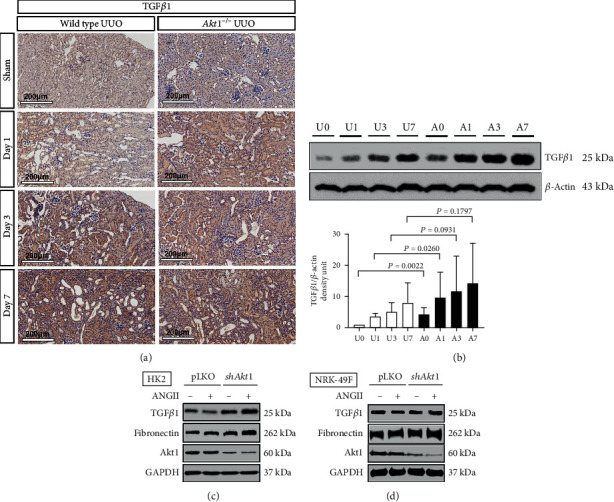
*Akt1* deletion is associated with upregulation of TGF*β*1. (a) Immunohistochemical staining (×200). Expression of TGF*β*1 began to increase from day 1 after UUO in *Akt1*^−/−^ mice. TGF*β*1 was expressed more strongly in *Akt1*^−/−^ mice than in wild type mice as UUO progressed. (b) Western blot analysis. TGF*β*1 expression was higher in *Akt1*^−/−^ mice than in wild type mice on day 0 (U0 vs. A0, *P* = 0.0022) and day 1 (U1 vs. A1, *P* = 0.0260) but was not statistically different between the two groups on day 3 (U3 vs. A3, *P* = 0.0931) and day 7 (U7 vs. A7, *P* = 0.1797). (c) Western blot analysis. In HK2 cells, silencing of *Akt1* promoted the expression of TGF*β*1 and fibronectin in the unstimulated state, which was augmented by angiotensin II stimulation (1 *μ*M). (d) Western blot analysis. In NRK-49F cells, silencing of *Akt1* did not affect the expression of TGF*β*1 and fibronectin regardless of angiotensin II stimulation. Abbreviations: ANGII: angiotensin II; GAPDH: glyceraldehyde 3-phosphate dehydrogenase; HK2 cells: immortalized human proximal tubular cells; NRK-49F cells: rat kidney fibroblasts; TGF*β*1: transforming growth factor *β*1; UUO: unilateral ureteral obstruction; A0 (*n* = 6): *Akt1*^−/−^ sham; A1 (*n* = 6): day 1 *Akt1*^−/−^; A3 (*n* = 6): day 3 *Akt1*^−/−^; A7 (*n* = 6): day 7 *Akt1*^−/−^; U0 (*n* = 6): wild type sham; U1 (*n* = 6): day 1 wild type; U3 (*n* = 6): day 3 wild type; U7 (*n* = 6): day 7 wild type.

**Figure 4:**
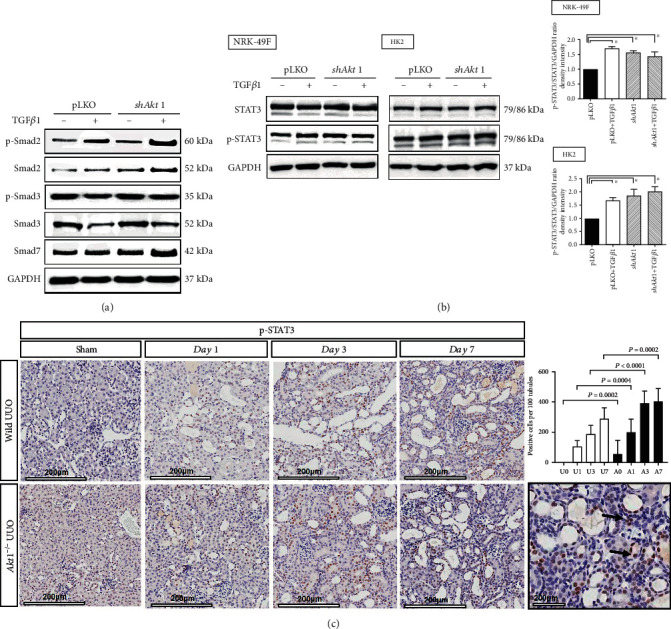
*Akt1* deletion is associated with activation of STAT3 independently of the Smad pathway. (a) Western blot analysis. In NRK-49F cells, there were no differences in the expression of phosphorylated- (p-) Smad2/3 and Smad7 both in pLKO and shAkt1 groups irrespective of TGF*β*1 treatment (10 ng/mL). (b) Western blot analysis. In NRK-49F and HK2 cells, p-STAT3/STAT3 ratios were significantly elevated in the pLKO group treated with TGF*β*1 and the shAkt1 group compared with the pLKO group not treated with TGF*β* (^∗^*P* < 0.05). (c) Immunohistochemical staining. After UUO, the expression of p-STAT3 was more increased in Akt1^−/−^ mice compared to wild type mice even from day 0 (×200). Picture surrounded by a black box shows the pattern of expression of p-STAT3 in tubular cells and surrounding cells in day 7 Akt1^−/−^ kidney (×400, black arrows: brown color-stained cells). Abbreviations: GAPDH: glyceraldehyde 3-phosphate dehydrogenase; NRK-49F cells: rat kidney fibroblasts; STAT3: signal transducer and activator of transcript 3; TGF*β*1: transforming growth factor *β*1; UUO: unilateral ureteral obstruction; A0 (*n* = 6): *Akt1*^−/−^ sham; A1 (*n* = 6): day 1 *Akt1*^−/−^; A3 (*n* = 6): day 3 *Akt1*^−/−^; A7 (*n* = 6): day 7 *Akt1*^−/−^; U0 (*n* = 6): wild type sham; U1 (*n* = 6): day 1 wild type; U3 (*n* = 6): day 3 wild type; U7 (*n* = 6): day 7 wild type.

After UUO surgery, the obstructed kidneys in both wild type and *Akt1*^−/−^ mice showed the typical features of obstructive nephropathy including tubular dilatation and renal tubulointerstitial fibrosis as evidenced by Masson's trichrome staining. The obstructed kidneys of the *Akt1*^−/−^ mice had more sclerotic area than kidneys of the wild type mice, especially in the earlier stages such as day 1 (U1 vs. A1, *P* = 0.022) and day 3 (U3 vs. A3, *P* = 0.041) (Figure 1(a)). The expression of fibronectin and type I collagen was significantly higher on day 3 (U3 vs. A3; fibronectin, *P* = 0.022; type I collagen, *P* = 0.031) and day 7 (U7 vs. A7; fibronectin, *P* = 0.041; type I collagen, *P* = 0.002). The expression of fibronectin and type I collagen was especially high around the damaged renal tubules.  Figure 2 shows the results of western blot analysis for proteins associated with tubulointerstitial fibrosis in *Akt1*^−/−^ mice and wild type mice. Akt1 expression was increased after UUO in wild type mice compared to wild type sham mice, while Akt1 was rarely expressed in the *Akt1*^−/−^ mice (Figures 2(a) and 2(b), (1)). Minimal expression of Akt1 in *Akt1*^−/−^ mice was thought to be due to nonspecific binding of the Akt1 antibody because the deletion of the Akt1 gene was confirmed by genotyping of the tail DNA. Next, we investigated the expression of Akt2 in the two murine models. The expression of Akt2 was increased to a similar degree in both the groups after UUO independent of Akt1 deletion (Figures 2(a) and 2(b), (2)). The expression of fibronectin and type 1 collagen increased gradually during UUO progression in wild type mice and *Akt1*^−/−^ mice compared to wild type sham mice and *Akt1*^−/−^ sham mice, respectively. On day 7 after UUO, *Akt1*^−/−^ mice showed a higher level of fibronectin than wild type mice (Figures 2(a) and 2(b), (3)). Type I collagen was also more strongly expressed in *Akt1*^−/−^mice than in wild type mice on days 1, 3, and 7 after UUO (Figures 2(a) and 2(b), (4)).

### 3.2. The Effect of Akt1 Deletion on TGF*β*1 Expression In Vivo and In Vitro

The analysis of IHC images (Figure 3(a)) revealed that TGF*β*1 was expressed more strongly in the *Akt1*^−/−^ mice than in the wild type mice after UUO. Interestingly, the expression of TGF*β*1 began to increase in the tubulointerstitial area from day 1 after UUO in the *Akt1*^−/−^ mice, indicating that TGF*β*1 is induced sooner in the *Akt1*^−/−^ mice than in the wild type mice. The analysis of western blot data (Figure 3(b)) revealed that the expression of TGF*β*1 increased gradually in both *Akt1*^−/−^ and wild type mice as UUO progressed. Interestingly, TGF*β*1 was more highly expressed in the *Akt1*^−/−^ mice than in the wild type mice on day 0 (U0 vs. A0, *P* = 0.0022) before UUO. After UUO, the expression of TGF*β*1 was higher in the *Akt1*^−/−^ mice than in the wild type mice on day 1 (U1 vs. A1, *P* = 0.0260) but was not statistically different between the two groups on day 3 (U3 vs. A3, *P* = 0.0931) and day 7 (U7 vs. A7, *P* = 0.1797). In summary, the deletion of *Akt1* induced rapid and intense upregulation of TGF*β*1 even from day 0 before UUO, highlighting the close relationship between Akt1 and TGF*β*1 (Figure 3(b)).

Angiotensin II and TGF*β*1 have been regarded as major culprits of renal fibrosis in UUO. Thus, we conducted an in vitro study using angiotensin II in two specific kidney cells, namely, HK2 cells and NRK-49F cells. In HK2 cells, the knockdown of *Akt1* promoted the expression of TGF*β*1 in the unstimulated state, which was augmented by angiotensin II stimulation (Figure 3(c), a 2.8-fold increase in optical density, *P* = 0.026). However, in NRK-49F cells, the silencing of *Akt1* did not affect the expression of TGF*β*1 regardless of angiotensin II stimulation (Figure 3(d)*P* = 0.66). Next, we investigated the fibronectin expression after angiotensin II stimulation. In HK2 cells, the knockdown of Akt1 enhanced the expression of fibronectin in the unstimulated state, which was augmented by angiotensin stimulation (Figure 3(c)). However, in NRK-49F cells, the silencing of Akt1 did not affect the expression of fibronectin regardless of angiotensin II stimulation (Figure 3(d)).

Then, we performed another in vitro study to investigate whether the TGF*β*1-related canonical pathway, the Smad pathway, is associated with genetic silencing of *Akt1*. In NRK-49F cells, there was no difference in the expression of p-Smad2/3 and Smad7 in both pLKO and *shAkt1* cells irrespective of TGF*β*1 treatment (Figure 4(a)). However, the ratio of p-STAT3/STAT3 was significantly increased in the pLKO group treated with TGF*β*1 compared to the pLKO cells not treated with TGF*β*1 (Figure 4(b)) in NRK-49F and HK2 cells. Irrespective of TGF*β*1 treatment, the ratios of p-STAT3/STAT3 were higher in the *shAkt1* group than in the pLKO group not treated with TGF*β*1 (Figure 4(b)). Consistent with in vitro results, p-STAT3 expression was more increased in *Akt1*^−/−^ mice compared to wild type mice in IHC analysis. Interestingly, loss of the Akt1 gene itself induced p-STAT3 expression (U0 vs. A0, *P* = 0.0002) (Figure 4(c)).

### 3.3. The Effect of the Akt1 Deletion on Apoptosis In Vivo and In Vitro

**Figure 5:**
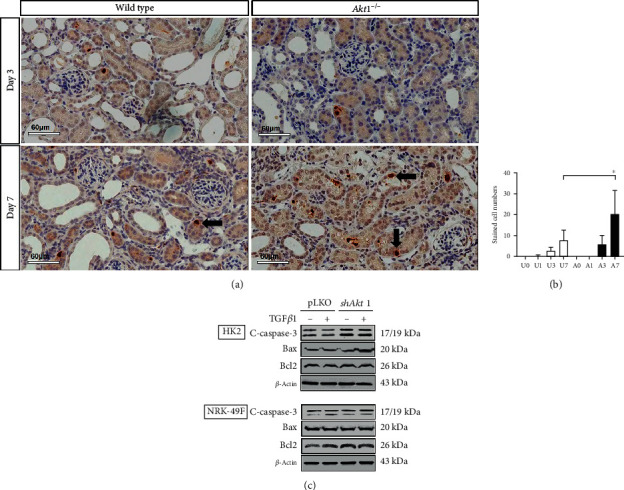
*Akt1* deletion promotes apoptosis. (a) Immunohistochemical staining. After UUO, cleaved- (c-) caspase-3 was expressed strongly in the nuclei of the renal tubular epithelial on day 7 in both *Akt1*^−/−^ and wild type mice (black arrow) (×400). (b) Quantification of stained cells for c-caspase-3. Expression of c-caspase-3 was significantly higher in *Akt1*^−/−^ mice than in wild type mice on day 7. (c) Western blot analysis. In HK2 cells, knockdown of *Akt1* led to increased expression of c-caspase-3 in the unstimulated state, which remained elevated upon TGF*β*1 stimulation (10 ng/mL). Bax also increased its expression by TGF*β*1 stimulation in the *shAkt1* group with cleaved caspase-3. In NRK-49F cells, knockdown of *Akt1* did not affect the expression of c-caspase-3 regardless of TGF*β*1 stimulation. Abbreviations: HK2 cells: immortalized human proximal tubular cells; NRK-49F cells: rat kidney fibroblasts; TGF*β*1: transforming growth factor *β*1; UUO: unilateral ureteral obstruction; A0 (*n* = 6): *Akt1*^−/−^ sham; A1 (*n* = 6): day 1 *Akt1*^−/−^; A3 (*n* = 6): day 3 *Akt1*^−/−^; A7 (*n* = 6): day 7 *Akt1*^−/−^; U0 (*n* = 6): wild type sham; U1 (*n* = 6): day 1 wild type; U3 (*n* = 6): day 3 wild type; U7 (*n* = 6): day 7 wild type. ^∗^*P* < 0.05.

The IHC data revealed that the expression of cleaved caspase-3 was significantly higher in *Akt1*^−/−^ mice than in wild type mice on day 7 after UUO (Figures 5(a) and 5(b)). In HK2 cells, silencing of *Akt1* led to an increase in the expression of cleaved caspase-3 in the unstimulated state; the expression remained elevated upon TGF*β*1 stimulation. Bax also increased its expression by TGF*β*1 stimulation in the *shAkt1* group with cleaved caspase-3. In NRK-49F cells, silencing of *Akt1* did not affect the expression of cleaved caspase-3 and Bax regardless of TGF*β*1 stimulation (Figure 5(c)). This finding implies that Akt1 might be related to renal tubular epithelial apoptosis as well as tubulointerstitial fibrosis.

## 4. Discussion

CKD is a syndromic disease characterized by diminished renal function, which microscopically manifests as glomerulosclerosis and tubulointerstitial fibrosis combined with tubular apoptosis. This study was aimed at investigating the role of Akt1 in tubular apoptosis and tubulointerstitial fibrosis for which the UUO murine model was selected. UUO model is a well-established model of CKD leading to tubulointerstitial fibrosis with distinct patterns of cell proliferation and apoptosis in the obstructed kidneys [14]. Both tubulointerstitial fibrosis and apoptosis induce loss of renal mass and renal dysfunction in obstructive nephropathy [14]. Additionally, we tested the effect of genetic silencing of *Akt1* in two cell lines (proximal renal tubular cells and renal fibroblasts) to verify the association between Akt1 and TGF*β*1 expression in tubular apoptosis.

In previous studies, the role of Akt in tubulointerstitial fibrosis and tubular cell apoptosis was demonstrated in an animal model of UUO [15, 16]. PI3K/Akt activity was found to be increased in ligated kidneys compared to that in nonligated kidneys [15]. Treatment with the PI3K inhibitor, Ly294002, suppressed the UUO-induced tubulointerstitial fibrosis as evidenced by decreased levels of fibroblast markers and extracellular matrix deposition in the interstitium [16]. Ly294002 treatment also reduced the number of proliferating cells in the interstitium and tubules [16]. However, which of the three Akt isoforms are associated with tubulointerstitial fibrosis remains poorly understood. Previously, we reported that genetic silencing of the Akt1 isoform caused the vascular smooth muscle cells to switch from the contractile to the synthetic phenotype [12]. Based on these previous studies, we hypothesized that the Akt1 isoform might be associated with renal tubulointerstitial fibrosis.

The principal findings of the current study are that genetic deletion of *Akt1* could aggravate kidney fibrosis in murine models of UUO, which may be related to the activation of the TGF*β*1/STAT3 pathway. As we discussed above, increased Akt activity has been reported in experimental tubulointerstitial fibrosis. Thus, there is a possibility that compensatory increase in the levels of other Akt isoforms (Akt2 or Akt3) could contribute to tubulointerstitial fibrosis and tubular atrophy in *Akt1*^−/−^ mice. As it is known that Akt3 protein is not present in the kidneys [17], we investigated the expression of Akt2 in obstructed kidneys of both *Akt1*^−/−^ and wild type mice. Akt2 expression did not differ between the two murine models, indicating that the promotion of tubulointerstitial fibrosis in *Akt1*^−/−^ mice is primarily associated with the deletion of *Akt1* and not with the compensatory increase in the level of Akt2.

Although the expression of TGF*β*1 increased gradually in the murine models after UUO, *Akt1*^−/−^ mice showed higher expression of TGF*β*1 than wild type mice. Interestingly, *Akt1*^−/−^ mice showed more rapid and intense upregulation of TGF*β*1 (even from day 0 before UUO) than the wild type mice, highlighting the close relationship between Akt1 and TGF*β*1. The degree of TGF*β*1 expression in *Akt1*^−/−^ mice on day 1 after UUO was similar to that observed in wild type mice on day 7 after UUO. Furthermore, in vivo results were confirmed by in vitro experiments, which showed that knockdown of *Akt1* induced TGF*β*1 in proximal tubular cells, but not in fibroblasts. We also investigated which one of the TGF*β*1 signaling pathways was associated with the genetic deletion of *Akt1*. TGF*β*1 plays a pivotal role in the pathogenesis of renal fibrosis [18, 19]. TGF*β*1 exerts its pathological activities via Smad-dependent and Smad-independent signaling pathways [18, 19]. In the Smad-dependent pathway, the binding of TGF*β*1 to its receptor leads to phosphorylation, whereas in the Smad-independent pathway, TGF*β*1 utilizes multiple signaling pathways including the MAPK pathway, rho-like GTPase signaling pathway, and JAK-STAT pathway [18, 19]. In this study, the expression levels of p-Smad2/3 were not different between the control and *Akt1*^−/−^ NRK-49F cells irrespective of TGF*β*1 treatment. However, the ratio of p-STAT3/STAT3 was significantly elevated in the sh*Akt1* groups NRK-49F and HK2 cells compared to the control groups not treated with TGF*β*1. Consistent with in vitro results, IHC analysis showed that p-STAT3 expression was more increased in *Akt1*^−/−^ mice compared to wild type mice even from day 0 before UUO. These results suggest that the genetic deletion of *Akt1* might be associated with the activation of STAT3 independently of the Smad signaling pathway. Indeed, a previous study showed that PI3K/AKT inhibition induces compensatory activation of STAT3 in non-small cell lung cancer [20]. Taken together, although further studies are needed to confirm the results of our study, we suggest that TGF*β*1 upregulation upon genetic deletion of *Akt1* may contribute to kidney fibrosis via the upregulation of STAT3.

Along with kidney fibrosis, apoptosis is also an important pathogenetic factor of CKD [18, 21, 22]. TGF*β*1 contributes to apoptosis in various types of kidney cells including the tubular epithelial cells during kidney fibrosis and glomerulosclerosis [22]. In the regulation of cell survival, Akt has been reported to block apoptosis through phosphorylation of multiple substrates involved in apoptosis [6]. However, the role of Akt in apoptosis during renal tubulointerstitial fibrosis is not well known. In the current study, we found that the expression of cleaved caspase-3 was significantly higher in the *Akt1*^−/−^ mice than in the wild type mice, and the expression was confined to nuclei of the renal tubular epithelial cells. Consistent with the results of in vivo experiments, we found that knockdown of *Akt1* increased the expression of cleaved caspase-3 and Bax in proximal tubule cells, but not in fibroblasts. Recently, a skeletal muscle-specific Akt1 transgenic (Akt1 TG) mouse can prevent the skeletal muscle loss by UUO, and its effect mitigated the renal fibrosis within the kidney, in which Akt1 was preserved [23]. A skeletal muscle-specific Akt1 TG was not a similar situation with our murine model, systemic Akt1 KO. Generally, the role of Akt1 is not uniform but various and finely tuned according to cellular or organ stimulation. In our results, the effect of the skeletal muscle cannot be excluded. In other words, our murine models could not preserve the skeletal musculature by systemic Akt1 KO; thus, this skeletal loss by Akt1 KO could contribute to kidney injuries including fibrosis and apoptosis. We thought that it would be necessary to uncover the process in the future in terms of interorgan connection.

Akt is a survival protein that participates in many intracellular processes [7, 12]. Although Akt can be traditionally activated through a PI3K-dependent pathway, it has also been reported to be activated via a PI3K-independent pathway and to act in a disease- and cell type-dependent manner [24]. Thus, we considered that in vivo or in vitro models of Akt isoform-specific genetic deletion are needed to explore the biological functions of Akt isoforms in kidney fibrosis. From this point of view, our results may guide future research on this subject.

## 5. Conclusions

The genetic deletion of Akt1 promoted the UUO-induced TGF*β*1 expression, fibrosis marker expression, and tubular apoptosis in vivo. In vitro, we confirmed that the genetic silencing of Akt1 induced the TGF*β*1 expression and apoptosis in proximal tubule cells, not in fibroblasts. We also showed that TGF*β*1 upregulation by genetic deletion of Akt1 is associated with activation of STAT3 independently of the TGF*β*1/Smad signaling pathway. Taken together, our findings suggest that the deletion of *Akt1* could aggravate kidney fibrosis and tubular cell apoptosis through activation of the TGF*β*1/STAT3 pathway in the murine model of UUO.

## Data Availability

The data used to support the findings of this study are included within the article.
